# Association between Serum Liver Enzymes and Metabolic Syndrome in Korean Adults

**DOI:** 10.3390/ijerph15081658

**Published:** 2018-08-05

**Authors:** Hae Ran Kim, Mi Ah Han

**Affiliations:** 1 Department of Nursing, School of Medicine, Chosun University, Gwangju 61452, Korea; rahn00@chosun.ac.kr; 2 Department of Preventive Medicine, School of Medicine, Chosun University, Gwangju 61452, Korea

**Keywords:** alanine transaminase, aspartate aminotransferase, health surveys, metabolic syndrome

## Abstract

The purpose of this study was to evaluate the association between alanine aminotransferase (ALT) and aspartate aminotransferase (AST), and metabolic syndrome (MS) in the Korean population. A total of 11,587 adults ≥30 years of age and with complete data were selected from Korea National Health and Nutrition Examination Survey VI conducted from 2013 to 2015. Normal ALT and AST levels were divided into quartiles, and their associations with MS were assessed by logistic regression analysis. Elevated levels of ALT [adjusted odds ratio (aOR) = 7.90, 95% confidence interval (CI) = 6.50–9.60] and AST (aOR = 3.81, 95% CI = 3.10–4.74) were both significantly associated with a higher prevalence of MS. The quartile group containing the highest levels of ALT and AST within the normal range also showed an increased risk of MS and its components. Elevated levels of ALT and AST, even within the normal range, were associated with prevalence of MS.

## 1. Introduction

Alanine aminotransferase (ALT) and aspartate aminotransferase (AST) are liver enzymes that are commonly used for liver function blood tests. Elevation of ALT and AST levels is an indicator of liver damage [1]. Recent studies have shown that elevated levels of serum aminotransferases are associated with medical conditions such as dementia, stroke, colorectal adenoma, frailty, disability, sarcopenia, metabolic syndrome (MS), and liver injury [2,3,4]. MS is a combination of risk factors such as high blood glucose, obesity, hypertension, and dyslipidemia, which are important predictors of cardiovascular disease and diabetes [5].

The prevalence of MS has steadily increased in Korea. The age-adjusted prevalence of MS was 24.9% in 1998, 29.2% in 2001, 30.4% in 2005, and 31.3% in 2007, using the data from Korea National Health and Nutrition Examination Survey (KNHANES) [6]. Age, smoking, alcohol consumption, income, family history of disease, and physical activity were associated with MS [7,8]. In Korea, age, smoking, alcohol drinking, and socioeconomic status were associated with MS [9], and lifestyle risk factors such as smoking and drinking were clustered in subjects with MS [10].

Studies have demonstrated the association of MS with elevated ALT and AST levels [11,12,13,14]. ALT and the AST-to-ALT ratio were significantly associated with risk of incident MS in a prospective cohort study among subjects aged 40–69 years in the USA [13]. Adults with MS have a significantly higher prevalence of unexplained elevations in ALT level using the US National Health and Nutrition Examination Survey (NHANES) [14]. Elevated liver enzymes within normal range were also associated with metabolic conditions among adults aged 45–75 years in Germany [15] and the mean concentrations of ALT increased significantly with increasing the number of metabolic abnormalities within normal range of liver enzymes in Iranian adults [11].

The elevated liver enzymes can be regarded as a surrogate marker of non-alcoholic fatty liver disease (NAFLD) which is liver fat accumulation [16]. A similar prevalence pattern has been reported in NAFLD and MS [17] and both were associated with dyslipidemia, central obesity, and insulin resistance [11]. Therefore, NAFLD has been regarded as a possible component in the cluster of MS [18].

In Korea, elevated liver enzymes were associated with MS. ALT and the AST-to-ALT ratio were associated with clustering of MS components in Korean adolescents [19]. Serum ALT levels were correlated with the component of MS in Korean postmenopausal women [20]. However, data for assessing the association between elevated liver enzymes within normal range and MS were limited in Korea. Thus, we investigated the relationship between elevated ALT/AST levels and MS based on data from the sixth KNHANES. The difference of MS according to the level of liver enzymes within normal range was investigated. 

## 2. Methods

### 2.1. Data Source

This study utilized data from the sixth KNHANES, which was conducted from 2013 to 2015 by the Korean Centers for Disease Control and Prevention. The purpose of this cross-sectional survey is to help establish national health policies and develop knowledge bases for the assessment of health risk factors and health indicators. The KNHANES used two-stage probability sampling methods to select nationally representative samples of non-institutionalized civilians and evaluated health status and related behavior at the national level. First, 576 (192 per year) primary sampling units (PSUs) were sampled based on administrative districts and housing types. Then, systematic sampling was applied to select 20 households from each PSU after excluding facilities such as nursing homes, military facilities, prisons, and foreign households. All individuals aged one year and over in selected households were selected as study subjects. KNHANES VI consisted of health interviews, health examinations, and nutrition surveys. Data from the health interview and health examination were used in this study. All health interviews and examinations were performed by trained staff members and all equipment was calibrated periodically [21]. From a total of 22,948 participants, the subjects who were unable to be evaluated for MS were excluded, and 11,587 subjects who were over 30 years old and had undergone blood tests for ALT and AST levels were included in the final analysis.

### 2.2. Criteria of Metabolic Syndrome

MS was determined according to the unified criteria published in the National Cholesterol Education Program Adult Treatment Panel III guideline [22]. These include the following: (1) abdominal obesity, defined as waist circumference ≥90 cm for men and ≥85 cm for women; (2) fasting blood glucose levels ≥110 mg/dL; (3) triglycerides ≥150 mg/dL; (4) high-density lipoprotein (HDL) cholesterol <40 mg/dL for men, and <50 mg/dL for women; and (5) hypertension, defined as systolic blood pressure ≥130 mmHg or diastolic blood pressure ≥85 mmHg, and taking antihypertensive medication. If the subjects met three or more of these criteria, they were classified as having MS. 

Waist circumference was measured at the midpoint between the lower costal margin and the upper margin of iliac crest using a tape measure (Seca 220; Seca, Hamburg, Germany). Blood pressure was measured three times after 5 min rest using a mercury sphygmomanometer (Baumanometer; Baum, Copiague, NY, USA) and the average of the last two measurements was used. Venous blood samples after 8 h of fasting were collected and serum levels of glucose, triglycerides, and cholesterol were measured using a Hitachi Automatic Analyzer 7600 (Hitachi, Tokyo, Japan). 

### 2.3. Criteria for Alanine Aminotransferase and Aspartate Aminotransferase Levels

Fasting venous blood serum were collected and serum levels of ALT and AST were measured using a Hitachi Automatic Analyzer 7600 (Hitachi, Tokyo, Japan). Elevated serum ALT or AST levels were defined as greater than 40 U/L according to the conventional value [23,24]. Elevated liver enzymes (even if within normal range) were associated with metabolic disorders [11,15]. Thus, subjects within the normal range were divided into quartiles. Within the normal range, AST/ALT levels were divided into the following quartiles: ≤13 U/L, 14–17 U/L, 18–22 U/L, and 23–40 U/L for ALT; and ≤17 U/L, 18–20 U/L, 21–24 U/L, and 25–40 U/L for AST. 

### 2.4. Covariates

Variables associated with MS in previous studies were selected as covariates [8,9,10] and data from health interview surveys were used. Independent variables included age (continuous), sex (men, women), education (≤elementary school, middle school, ≥high school), current occupation (yes, no), marital status (living with spouse, others), household income (low, middle-low, middle-high, high), current smoking status (yes, no), current drinking status (yes, no), and physical activity (1–2, ≥3 days/week). Income was divided into quartiles based on the data of total household income.

### 2.5. Statistical Analysis

Data were analyzed using IBM SPSS Statistics for Windows, version 24 (IBM Corp., Armonk, NY, USA). Prevalence of MS and elevation of liver enzyme levels were analyzed by frequency analysis. The difference of age by MS was compared using *t*-test and the prevalence of MS by general characteristics were compared using Chi-squared tests. The relationship between MS components and the range of liver enzyme levels was analyzed using the Chi-square test. Finally, multiple logistic regression analyses were used to identify the odds ratios (ORs) for liver enzyme levels, which were used as independent variables to study the association of liver enzyme levels with MS and its components. As the data of KNHANES was acquired using stratified, clustered, and systematic sampling, we used analysis methods for complex samples. All results excepting number reported as weighted values. Differences were considered statistically significant at *p* < 0.05.

## 3. Results

### 3.1. Prevalence of Metabolic Syndrome

MS was prevalent in 26.9% of subjects. The prevalence of MS components are as follows: 28.7% for abdominal obesity, 32.9% for high blood pressure, 31.7% for high triglycerides, 35.9% for low HDL-cholesterol, and 37.3% for high blood glucose. Overall, 7.8% of the study subjects had elevated ALT levels, and 4.5% had elevated AST levels.

The prevalence of MS was higher in men (30.0%) than in women (24.6%) (*p* < 0.001), and higher in subjects with lower levels of education (35.8%) than those with higher levels of education (18.5%) (*p* < 0.001). MS prevalence was also higher in subjects without occupation (29.6%), in subjects with lower income (37.1%), and current smokers (31.2%) (*p* < 0.001) (Table 1).

### 3.2. Correlation between Liver Enzyme Levels and the Prevalence of Metabolic Syndrome and Its Components

MS was prevalent in 53.7% of subjects with elevated ALT levels. The prevalence of MS components in subjects with elevated ALT levels was as follows: 52.2% for abdominal obesity, 44.2% for high blood pressure, 57.2% for high triglycerides, 58.0% for low HDL-cholesterol, and 57.3% for high blood glucose. Within the normal range of ALT levels, the prevalence of MS, abdominal obesity, high blood pressure, high triglycerides, low HDL-cholesterol, and high blood glucose increased significantly when enzyme levels increased (*p* < 0.001). MS was prevalent in 49.8% of subjects with elevated AST levels. The prevalence of MS components in subjects with elevated AST levels was as follows: 50.2% for abdominal obesity, 49.9% for high blood pressure, 49.9% for high triglycerides, 39.1% for low HDL-cholesterol, and 58.8% for high blood glucose. Within the normal range of AST levels, the prevalence of MS, abdominal obesity, high blood pressure, high triglyceride, and high blood glucose also increased significantly with increasing enzyme levels (*p* < 0.001) (Table 2).

### 3.3. The Odds Ratios for Metabolic Syndrome and Its Components at Different Liver Enzyme Levels

Within the normal range of liver enzyme levels, the ORs of MS, and its components significantly increased in quartiles with higher levels of ALT and AST. In subjects with elevated levels of ALT, the OR of MS was 7.90 (95% confidence interval (CI) = 6.50–9.60), which was significantly higher than that of the subjects in normal quartile 1. The same applied to the OR of abdominal obesity (6.89, 95% CI = 5.56–8.52), high blood pressure (2.36, 95% CI = 1.86–3.00), high triglycerides (5.67, 95% CI = 4.71–6.83), low HDL-cholesterol (2.01, 95% CI = 1.63–2.47), and high blood glucose (3.99, 95% CI = 3.16–5.02). Similarly, in subjects with elevated AST levels, the OR of MS was 3.81 (95% CI = 3.10–4.74), which was significantly higher than that of the subjects in normal quartile 1. The OR of abdominal obesity (3.24, 95% CI = 2.55–4.12), high blood pressure (2.60, 95% CI = 2.10–3.22), high triglycerides (3.17, 95% CI = 2.51–4.01), and high blood glucose (2.42, 95% CI = 1.93–3.04) were also higher than those in the normal quartiles (Table 3).

## 4. Discussion

Many epidemiological studies have used ALT and AST levels as measures to assess fat accumulation in hepatocytes. Increased ALT levels were associated with a higher likelihood of obesity, hypertension, hypertriglyceridemia, elevated blood glucose, and cardiovascular disease [15,25,26]. Furthermore, recent studies have reported the association of serum ALT and AST levels with MS [27] and its components [15]. 

Elevated liver enzymes more than upper normal limits (>40 U/L) were associated with increased risk of MS in this study. Similar results were reported in previous studies using US NHANES. Elevated ALT levels (>40 U/L for men and >31 U/L for women) and AST levels (>37 U/L for men and >31 U/L for women) were associated with diabetes [28]. More than 40 U/L of ALT and AST were associated with low LDL and high HDL cholesterol [29].

Also, this study showed an increased prevalence of MS in the quartile group with the highest levels of ALT and AST within the normal range. The OR of MS and its components also increased with increasing ALT and AST levels. These results are consistent with previous studies. The prevalence of MS significantly increased from the first to the fifth quintile of ALT in the normal range [30]. Higher levels of ALT within the normal range were an independent risk factor for diabetes [1]. In addition to conventional clinical uses for indicate hepatic injury or fatty liver, our results suggested that elevated ALT and AST levels that are within the normal range may also be predictors of MS or cardiovascular disease. In this study, normal liver enzyme levels in the four quartiles were: ≤13 U/L, 14–17 U/L, 18–22 U/L, and 23–40 U/L for ALT; and ≤17 U/L, 18–20 U/L, 21–24 U/L, and 25–40 U/L for AST. These concentration ranges could be used as references for screening ALT and AST serum levels, which may help with early detection of MS and prevent its progression. However, additional studies are needed to determine the reference values of ALT and AST levels that accurately predict the presence of MS in the Korean population.

We used the conventional definition of elevated liver enzymes (more than 40 U/L). Elevated liver enzymes, even if within normal range, were associated with MS. These associations suggested that current normal limits of liver enzymes might not fully identify MS and the associated metabolic disorder. Several studies and guidelines have proposed upper limit of normal liver enzyme levels which are lower than conventional values and differ by gender [31]. Future studies are needed to reveal the association between elevated liver enzymes and MS according to gender specific upper limits of liver enzymes. 

The strengths of this study are as follows: an analysis of the most recent database to ensure accurate representation of the Korean population; a study of the association of MS with ALT and AST levels within their normal range; and analysis of the prevalence of MS in different characteristic groups of the population. This cross-sectional study could not reveal temporal relationships due to the nature of KNHANES. Further prospective studies are required to confirm the results of this study. In addition, liver enzymes were measured a single time and this can vary even within individuals. However single measurement of liver enzymes usually leads to non-differential misclassification compared to multiple measurements over time, which would probably lead to our results toward the null [32,33].

## 5. Conclusions

This is a nationally representative study of the relationship between serum liver enzymes and MS in the Korean population. Even within the normal range, increased ALT and AST levels were associated with the risk of MS and its components. This study may provide a basis for the development of clinical guidelines to address MS and its components in the Korean population.

## Figures and Tables

**Table 1:** Prevalence of metabolic syndrome according to the subjects’ characteristics.

Variables	Metabolic Syndrome		*p*
	No	Yes	
Age (years)	52.93 ± 0.30	58.85 ± 0.29	<0.001
Sex			<0.001
Men	3490 (70.0)	1474 (30.0)	
Women	5011 (75.4)	1612 (24.6)	
Education			<0.001
≤Elementary school	2828 (64.2)	1564 (35.8)	
Middle school	2768 (75.9)	881 (24.1)	
≥High school	2905 (81.5)	641 (18.5)	
Current occupation			<0.001
Yes	5236 (74.9)	1740 (25.1)	
No	3265 (70.4)	1346 (29.6)	
Marital status			<0.001
Living with spouse	6945 (74.6)	2336 (25.4)	
Others	1556 (67.0)	750 (33.0)	
Household income			<0.001
Low	1416 (62.9)	821 (37.1)	
Middle-low	2808 (71.6)	1090 (28.4)	
Middle-High	2267 (77.8)	657 (22.2)	
High	2010 (79.0)	518 (21.0)	
Current smoking status			0.001
Yes	1485 (68.8)	674 (31.2)	
No	7016 (74.1)	2412 (25.9)	
Current drinking status			<0.001
Yes	6002 (74.4)	2029 (25.6)	
No	2499 (70.2)	1057 (29.8)	
Physical activity			<0.001
1–2 days/week	5243 (71.3)	2104 (28.7)	
≥3 days/week	3258 (76.2)	982 (23.8)	

Values are expressed as mean ± standard error or frequency (%).

**Table 2:** The prevalence of metabolic syndrome and its components according to the liver enzyme.

**Variables**	**Normal ALT**					**ALT**		
	**Quartile 1** **(*n* = 3061)**	**Quartile 2** **(*n* = 2717)**	**Quartile 3** **(*n* = 2358)**	**Quartile 4** **(*n* = 2550)**	***p***	**Normal** **(*n* = 10,686)**	**Elevated** **(*n* = 901)**	***p***
Metabolic syndrome	409 (14.0)	577 (21.6)	663 (27.6)	958 (37.7)	<0.001	2607 (24.6)	479 (53.7)	<0.001
Abdominal obesity	449 (14.9)	608 (23.5)	713 (30.8)	1019 (40.2)	<0.001	2789 (26.7)	475 (52.2)	<0.001
High blood pressure	710 (23.8)	813 (30.6)	828 (35.0)	996 (40.4)	<0.001	3347 (32.0)	392 (44.2)	<0.001
High triglyceride	493 (16.2)	701 (26.4)	822 (34.9)	1132 (43.9)	<0.001	3148 (29.6)	513 (57.2)	<0.001
Low HDL-cholesterol	1033 (34.3)	972 (35.8)	829 (34.8)	956 (36.9)	<0.001	6896 (64.6)	525 (58.0)	0.003
High blood glucose	709 (23.5)	948 (34.8)	945 (39.0)	1231 (48.1)	<0.001	3833 (35.6)	506 (57.3)	<0.001
**Variables**	**Normal AST**					**AST**		
	**Quartile 1** **(*n* = 3057)**	**Quartile 2** **(*n* = 2741)**	**Quartile 3** **(*n* = 2705)**	**Quartile 4** **(*n* = 2542)**	***p***	**Normal** **(*n* = 11,045)**	**Elevated** **(*n* = 542)**	***p***
Metabolic syndrome	521 (16.9)	671 (24.7)	729 (28.0)	902 (35.1)	<0.001	2823 (25.8)	263 (49.8)	<0.001
Abdominal obesity	604 (20.4)	695 (25.7)	774 (29.2)	917 (36.6)	<0.001	2990 (27.7)	274 (50.2)	<0.001
High blood pressure	645 (21.8)	846 (31.1)	978 (36.7)	1006 (40.5)	<0.001	3475 (32.1)	264 (49.9)	<0.001
High triglyceride	621 (19.8)	817 (30.4)	893 (33.4)	1061 (41.7)	<0.001	3392 (30.9)	269 (49.9)	<0.001
Low HDL-cholesterol	1151 (37.1)	990 (36.1)	928 (35.2)	884 (34.7)	0.374	3953 (35.8)	213 (39.1)	0.218
High blood glucose	890 (28.9)	967 (35.0)	1048 (38.6)	1121 (44.0)	<0.001	4026 (36.3)	313 (58.8)	<0.001

AST: aspartate aminotransferase; ALT: alanine aminotransferase; HDL: high-density lipoprotein. Values are expressed as frequency (%).

**Table 3:** Odds ratios (95% confidence interval) for metabolic syndrome and components by the level of liver enzymes.

	**Normal ALT**				**Elevated ALT**	***p* for Trend**
**Variables**	**Quartile 1**	**Quartile 2**	**Quartile 3**	**Quartile 4**		
Metabolic syndrome	1.00	1.49 (1.28–1.74)	2.11 (1.82–2.45)	3.60 (3.10–4.19)	7.90 (6.50–9.60)	<0.001
Abdominal obesity	1.00	1.61 (1.41–1.85)	2.40 (2.10–2.75)	3.88 (3.34–4.51)	6.89 (5.56–8.52)	<0.001
High blood pressure	1.00	1.15 (1.00–1.33)	1.39 (1.21–1.60)	1.82 (1.57–2.10)	2.36 (1.86–3.00)	<0.001
High triglyceride	1.00	1.70 (1.45–1.99)	2.44 (2.08–2.86)	3.38 (2.96–3.86)	5.67 (4.71–6.83)	<0.001
Low HDL-cholesterol	1.00	1.10 (0.97–1.26)	1.15 (1.00–1.34)	1.44 (1.28–1.63)	2.01 (1.63–2.47)	<0.001
High blood glucose	1.00	1.40 (1.20–1.64)	1.67 (1.46–1.91)	2.46 (2.13–2.85)	3.99 (3.16–5.02)	<0.001
	**Normal AST**				**Elevated AST**	***p* for trend**
**Variables**	**Quartile 1**	**Quartile 2**	**Quartile 3**	**Quartile 4**		
Metabolic syndrome	1.00	1.37 (1.19–1.57)	1.49 (1.31–1.70)	2.00 (1.73–2.31)	3.81 (3.10–4.74)	<0.001
Abdominal obesity	1.00	1.18 (1.01–1.39)	1.33 (1.15–1.53)	1.80 (1.58–2.05)	3.24 (2.55–4.12)	<0.001
High blood pressure	1.00	1.29 (1.12–1.48)	1.48 (1.25–1.74)	1.66 (1.40–1.96)	2.60 (2.10–3.22)	<0.001
High triglyceride	1.00	1.67 (1.44–1.93)	1.81 (1.58–2.01)	2.41 (2.10–2.77)	3.17 (2.51–4.01)	<0.001
Low HDL-cholesterol	1.00	0.93 (0.81–1.05)	0.89 (0.77–1.03)	0.92 (0.81–1.04)	1.17 (0.90–1.51)	<0.001
High blood glucose	1.00	1.03 (0.92–1.15)	1.05 (0.95–1.17)	1.23 (1.08–1.41)	2.42 (1.93–3.04)	<0.001

AST: aspartate aminotransferase; ALT: alanine aminotransferase; HDL: high-density lipoprotein. Adjusted for age, sex, education, current occupation, marital status, family income, current smoking status, current drinking status, and physical activity.
